# International cooperation through big science programs

**DOI:** 10.1093/nsr/nwac187

**Published:** 2022-09-07

**Authors:** Mu-ming Poo

**Affiliations:** Scientific Director, CAS Center for Excellence in Brain Science and Intelligence Technology, China; Executive Editor-in-Chief of NSR

Despite the COVID-19 pandemic and travel restrictions, scientists remain busy in forging international collaborations. Driven by the spirit of academic freedom in the pursuit of knowledge and the tradition of international collaboration, scientists from 16 countries have recently announced, at BGI-Research (previously known as Beijing Genomics Institute) in Shenzhen, the establishment of the Spatiotemporal Omics Consortium (STOC), an open collaborative research initiative to unite, advance and share global scientific efforts to solve the mysteries of life at genetic, molecular and cellular levels through large-scale spatiotemporally resolved multiomics analyses (https://www.sto-consortium.org/).

The most celebrated big science project in biology is the Human Genome Project (HGP), which was first initiated by US scientists and later grew to become an international consortium consisting of laboratories from the United States, United Kingdom, France, Germany, China and Japan. The data provided by the HGP contributed greatly to the rapid advances in biological sciences and biotechnology in the past two decades. This has inspired many subsequent big science projects aimed at characterizing the compositions of all mRNAs (transcriptome) and all proteins (proteome) in various cell types or tissues, the complete pattern of epigenetic modifications of genomic DNA (epigenome), and the wiring diagram of nerve connections in the entire brain (connectome).

In the dynamic open systems of cells and tissues, molecular components undergo continuous spatiotemporal changes that both reflect and affect biological processes in normal and disease states. Patterns of single-cell transcriptomes are now widely used for classifying cell types and states, and new technologies of single-cell spatial transcriptomics further allow the mapping of spatial distributions of cells expressing different transcriptomes (Ao *et al*. *Cell* 2022; doi:10:1016/j.cell.2022.04.003). Integration and understanding of multiomic data will provide new insights into the physiology, development and aging, disease pathogenesis, and evolutionary processes. The goals of STOC pose major scientific and technical challenges far exceeding those of the HGP and require large-scale, cross-disciplinary and international collaborations.

International big science programs have recently appeared in other fields. Deep-Time Digital Earth (DDE), the first big science program recognized by the International Union of Geological Sciences (IUGS), officially kicked off in Beijing in February 2019. First initiated by geologists from China and other countries, DDE has attracted more than 20 founding members from national geological surveys, international associations, academic institutions and research organizations. An overview of DDE projects will be provided in the forthcoming forum to be convened at UNESCO, Paris, on 9 November 2022.

The mission of DDE is to harmonize and share ‘deep-time’ digital geological data related to the changes of the Earth over millions of years of geological time, to develop and disseminate advanced methods for analyzing and visualizing geological data, and to promote data-driven research paradigms. These data address the evolution of life and climate, tectonic plate movement, and the evolution of the planet's geography, and will be made available to scientists in easily accessible ‘hubs’ to help achieve sustainable development goals (Wang *et al*. *NatlSciRev* 2021; doi:10.1093/nsr/nwab027).

In the big data era, both STOC and DDE are taking full advantage of new advances in information science, including big data analytics, internet cloud computing, data storage and mining, machine learning and other AI methods. While setting up the mechanisms for data sharing and dissemination remains challenging, the principle of openness and fairness, as stipulated by UNESCO’s Open Science Recommendation 2021, is endorsed by both STOC and DDE. Indeed, the attraction of big science programs comes from their platforms, through which collaborations could be effectively established for sharing technologies and data, and for making major scientific breakthroughs that are difficult or impossible to achieve by individual laboratories. For scientists preferring to work alone, the platform's openness will allow access to data and resources for individual scientists to explore their own line of research.

‘Science has no national boundary’ is not just a slogan, it is a tradition embedded in the hearts and minds of scientists around the world. International conferences, many of which have taken the form of online meetings due to COVID-19-related travel restrictions, have continued to take place in all fields in the past two years, symbolizing academic freedom and international cooperation with regard to solving critical issues of global concern. The establishment of international big science programs such as STOC and DDE offers the opportunity for scientists to work together effectively in order to achieve scientific progress that benefits all societies around the world.

